# Single Line-to-Ground Fault Type Multilevel Classification in Distribution Network Using Realistic Recorded Waveform

**DOI:** 10.3390/s23218948

**Published:** 2023-11-03

**Authors:** Jiajun Liu, Chenjing Li, Yue Liu, Ji Sun, Haokun Lin

**Affiliations:** School of Electrical Engineering, Xi’an University of Technology, Xi’an 710048, China

**Keywords:** single line-to-ground fault, fault recorder data, multi-type faults, multilevel classification

## Abstract

The further identification of fault types for single line-to-ground faults (SLGFs) in distribution networks is conducive to determining the cause of grounding faults and formulating targeted measures for hidden danger treatment and fault prevention. For the six types of SLGFs generated in the actual power grid, this paper deeply studies their fault characteristics. Firstly, the classification criterion of fault transition resistance is derived by the generation mechanism of fault zero sequence voltage (ZSV). At the same time, by comparing and analyzing the same and different characteristics between faults, three criteria for fault classification are obtained. Based on the above four criteria, a multilevel and multicriteria fault classification method is proposed to judge six types of SLGFs. Then, the proposed method is verified by various fault state simulations of the distribution network model with a balanced topology and unbalanced topology. The engineering application of the method is demonstrated by the verification of actual power grid data. Finally, noise and data loss interference test results show the robustness of the method.

## 1. Introduction

The probability of SLGFs in distribution network systems reaches 80% [1,2]. Due to the variety of fault types and working conditions, it is easy to fail in distinguishing fault types and performing troubleshooting in time, resulting in the expansion of the fault range. Therefore, it is a worthwhile task to further analyze the characteristics of multiple faults, as well as identifying and classifying the faults for the formulation of fault response measures in distribution networks.

There are various fault identification methods for distribution networks, mainly including fault feature analysis and data-driven artificial intelligence methods. On this basis, scholars have carried out extensive research on fault classification in distribution networks, including fault feeder detection, fault phase classification, and fault type classification.

Fault feeder detection and location: Most SLGF identification studies classify feeders into two categories, fault and health types, commonly referred to as SLGF feeder detection. Some studies use waveform analysis methods. In [3], the authors extracted the negative sequence component characteristics of a three-phase current and voltage through a wavelet transform (WT). Ref. [4] extracted the features by principal component analysis and obtained feeder discrimination results through the binary classification of the features using support vector machines. In [5], the zero sequence current (ZSC) matrix constructed by the Hilbert transform was decomposed by singular values to obtain an amplitude polarity eigenmatrix. There are also some studies using entropy [6], singular spectrum decomposition [7], feature mode decomposition [8], and so on. With the widespread application of artificial intelligence technology in power systems, some studies have used intelligent algorithms such as sparse encoders [9,10], machine learning [11,12,13], artificial neural networks [14,15,16], convolutional neural networks [17,18,19], and neural networks [20] for fault detection and location.

Fault phase classification: Research on multiple fault classification mainly focuses on the classification of grounding phases, aiming to distinguish whether a single line-to-ground fault, an interphase short-circuit fault, or a three-phase short-circuit fault has occurred. Ref. [21] used the depth map learning algorithm for fault location and classification. In [14], after extracting features with the Hilbert–Huang transform, a convolutional neural network (CNN) achieved the classification of the extracted features. Ref. [22] extracted the DC component via a mathematical morphology to realize the fault classification of power distribution cables.

Fault type classification: Recently, some researchers have emphasized the classification of fault types. Refs. [2,23,24] are classification studies aimed at identifying high-impedance faults. Refs. [25,26], respectively, applied the negative selection algorithm and CEEMDAN algorithm to distinguish the transition resistance degree in SLGFs. Ref. [27] describes a more comprehensive classification study of fault causes, using a dual-channel CNN intelligent algorithm. Ref. [28] focuses on two transient processes and detects intermittent grounding faults.

However, there are some problems in existing fault classification methods.

(1) Insufficient fault types: In the research of SLGF detection in a distribution network, there are few studies on fault type classification, and the types of faults are also limited. However, multiple types of faults occur in the actual operation of distribution networks, such as transition resistance grounding faults, arc grounding faults, intermittent grounding faults, and transient grounding faults. It is necessary to consider different types of faults, so as to formulate prevention and handling measures for specific faults.
(2) Classification method: The diversity of fault conditions and categories, as well as the limited applicability of a single identification method, can lead to misjudgment for different faults. Although artificial intelligence methods are often used to solve classification problems, they rely on the data size of the databases [29], especially for multi-fault classification research, posing challenges to the completeness of the database.
(3) Fault data: In actual distribution networks, most of the collected single line-to-ground fault data are recorded as SLGFs. These faults are not recognized as transition resistance grounding faults (TRGFs), arc grounding faults (AGFs), intermittent grounding faults (IGFs), transient grounding faults (TGFs), etc. Moreover, some faults, such as TGFs, may not require protection actions. Thus, it is difficult to obtain realistic recorded data containing multiple types of SLGFs.

The main contributions of this paper are as follows.

(1) Multi-type fault classification: We utilize six types of SLGFs in the actual distribution network to identify and classify the specific type of grounding fault, which is conducive to further determining the causes of grounding faults in the distribution network and formulating targeted measures for hidden danger management and fault elimination.
(2) Multilevel method: We construct a decision-tree-based multilevel strategy for fault type classification. By grouping the same features and distinguishing different features, four criteria are obtained based on the feature analysis of different fault types. Moreover, a multilevel progressive classification method for the SLGFs is carried out.
(3) Realistic recorded waveforms: The data verified in this paper are the actual single line-to-ground fault (SLGF) data in China’s distribution network, which include six types: small-impedance faults (SIFs), medium-impedance faults (MIFs), high-impedance faults (HIFs), arc grounding faults (AGFs), intermittent grounding faults (IGFs), and transient grounding faults (TGFs). The verification of the actual fault data reflects the engineering applicability of the method.

## 2. Fault Transition Conductance Analysis

**Figure 1:**
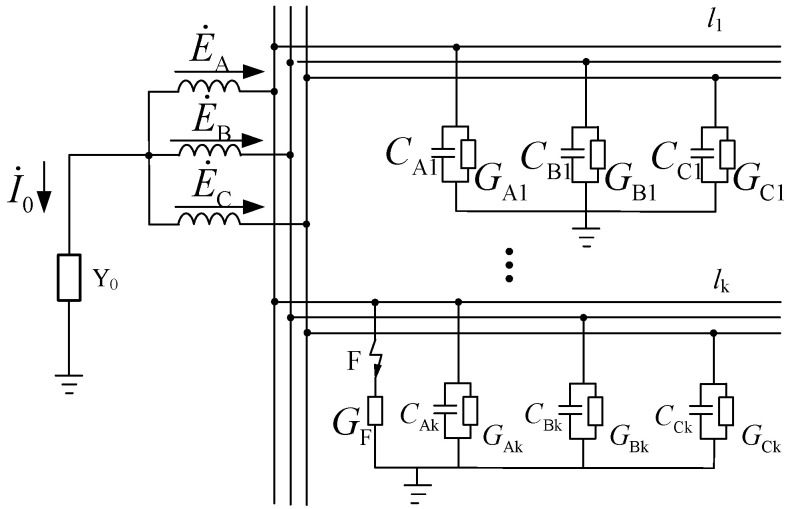
Equivalent operational circuit.

Figure 1 shows an equivalent operational circuit based on ZSV, where ${\dot{E}}_{A}$, ${\dot{E}}_{B}$, and ${\dot{E}}_{C}$ are the potentials of a three-phase power supply, $Y_{0}$ is the neutral grounding admittance, ${\dot{I}}_{0}$ is the ZSC flowing through the neutral branch, and $C_{\mathrm{Ak}}$ and $G_{\mathrm{Ak}}$ are the phase A to ground capacitance and conductivity of the feeder *l*_k_.

When a grounding fault has not yet occurred, the unbalanced current of feeder k is defined as ${\dot{I}}_{\mathrm{ubk}}$:

(1) $${\dot{I}}_{\mathrm{ubk}}={\dot{E}}_{A}Y_{\mathrm{Ak}}+{\dot{E}}_{B}Y_{\mathrm{Bk}}+{\dot{E}}_{C}Y_{\mathrm{Ck}}$$

Thus, the sum of the unbalanced currents of all feeders is

(2) $${\dot{I}}_{\mathrm{ub}\sum }=\left({\dot{E}}_{A}Y_{A}+{\dot{E}}_{B}Y_{B}+{\dot{E}}_{B}Y_{B}\right)$$

**Figure 2:**
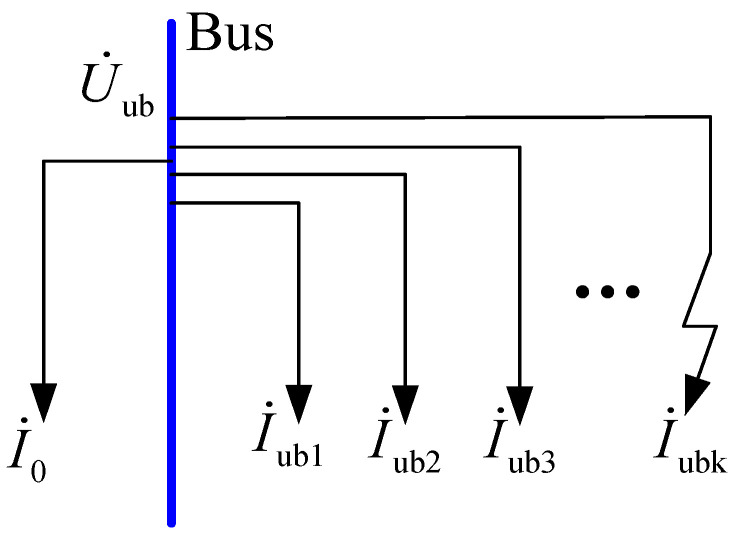
Unbalanced current of feeders.

As shown in Figure 2, in order to offset the unbalanced current of each feeder, ${\dot{U}}_{\mathrm{ub}}$ needs to be generated while ensuring that both parts of the neutral point comply with the Kirchhoff law. Adding the voltage ${\dot{U}}_{\mathrm{ub}}$ to the zero sequence impedance, the generated current is equal to the sum of all unbalanced currents, and Formula (3) is derived.

(3) $$-{\dot{I}}_{0}={\dot{I}}_{\sum \mathrm{ub}}={\dot{U}}_{\mathrm{ub}}\left(Y_{A}+Y_{B}+Y_{C}+Y_{0}\right)$$

When a single line-to-ground fault occurs in phase A, point F in Figure 1 is connected. $G_{F}$ is the transitional conductivity. The phase A to ground admittance becomes $Y_{A}{}^{\prime }=G_{F}+Y_{A}$, while the phase B and phase C to ground admittance remains unchanged. Thus, the total zero sequence admittance of the power grid is

(4) $$\begin{array}{cc} Y_{\sum } & =Y_{A}{}^{\prime }+Y_{B}+Y_{C}+Y_{0}\\ & =G_{F}+Y_{A}+Y_{B}+Y_{C}+Y_{0} \end{array}$$

The sum of the unbalanced currents during faults is

(5) $${\dot{I}}_{\mathrm{ub}\sum }{}^{\prime }=\left({\dot{E}}_{A}G_{F}+{\dot{E}}_{A}Y_{A}+{\dot{E}}_{B}Y_{B}+{\dot{E}}_{C}Y_{C}\right)$$

According to the ZSV generation principle in Figure 2, it can be obtained that

(6) $$-{\dot{I}}_{0}{}^{\prime }={\dot{I}}_{\mathrm{ub}\sum }{}^{\prime }={\dot{U}}_{\mathrm{ub}}{}^{\prime }\left(G_{F}+Y_{A}+Y_{B}+Y_{C}+Y_{0}\right)$$

By organizing Formulas (2) and (5), we obtain the unbalanced current relationship of pre- and post-fault.

(7) $${\dot{I}}_{\sum \mathrm{ub}}{}^{\prime }-{\dot{I}}_{\sum \mathrm{ub}}={\dot{E}}_{A}G_{F}$$

Using Formulas (3) and (6), it can be calculated from Formula (7) that

(8) $$\left({\dot{U}}_{\mathrm{ub}}{}^{\prime }-{\dot{U}}_{\mathrm{ub}}\right)\left(Y_{A}+Y_{B}+Y_{C}+Y_{0}\right)-{\dot{U}}_{A}G_{F}=0$$

Among them, ${\dot{U}}_{A}={\dot{U}}_{\mathrm{ub}}{}^{\prime }+{\dot{E}}_{A}$ is the voltage of the faulty phase, and $G_{F}$ can be further deduced.

(9) $$G_{F}=\frac{\left|{\dot{I}}_{F}\right|}{\left|{\dot{U}}_{A}\right|}=\frac{\left|{\dot{U}}_{\mathrm{ub}}{}^{\prime }-{\dot{U}}_{\mathrm{ub}}\right|\left(Y_{A}+Y_{B}+Y_{C}+Y_{0}\right)}{\left|{\dot{U}}_{A}\right|}$$

${\dot{I}}_{F}$ is the grounding fault current, indicating the current difference in the pre- and post-fault generated by the ZSV on the zero sequence admittance. From Formula (9), it can be concluded that the fault transition conductance is directly proportional to the variation of ZSV and inversely proportional to the fault phase voltage, which becomes the basis for Criterion 4 in Section 3.4.

## 3. Multilevel Classification Method

**Figure 3:**
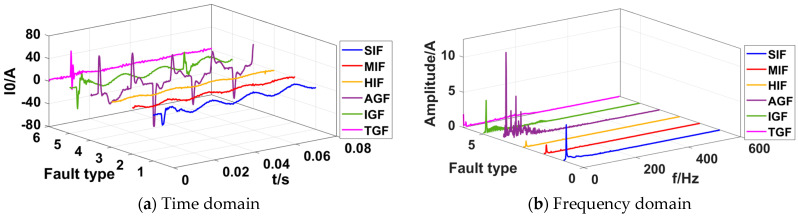
ZSC waveforms of 6 types of faults.

The fault recorder data of the Xinjiang region in 2021 are shown in Figure 3a, where the ZSC waveforms include the following six SLGFs: a small-impedance fault (SIF), medium-impedance fault (MIF), high-impedance fault (HIF), arc grounding fault (AGF), intermittent grounding fault (IGF), and transient grounding fault (TGF).

The diverse types of faults make it difficult to obtain a single criterion based on the characteristics of the zero sequence current waveforms. The FFT was used to transform the waveforms to the frequency domain, as shown in Figure 3b, and it can be seen that the frequency with the largest amplitude is still the fundamental frequency. Other high-frequency harmonics have extremely low amplitudes and a relatively small proportion, and they are relatively disorderly and irregular.

It can be seen that the criterion constructed by a single feature is insufficient to support the differentiation of multiple types of faults. To distinguish these 6 types of faults, progressive classification needs to be carried out based on their different features.

### 3.1. Criterion 1

Variational mode decomposition (VMD) differs in principle from empirical mode decomposition (EMD) and its improved algorithms, effectively avoiding modal aliasing. VMD decomposes sampled signal *X(t)* into several intrinsic mode functions (IMFs) with central frequencies [30]. The actual transient signal frequency of grounding faults in distribution networks is approximately 300 to 1000 Hz. Based on the decomposition test of actual fault-recorder waveforms, we found that it is appropriate to divide the waveforms into three modes.

**Figure 4:**
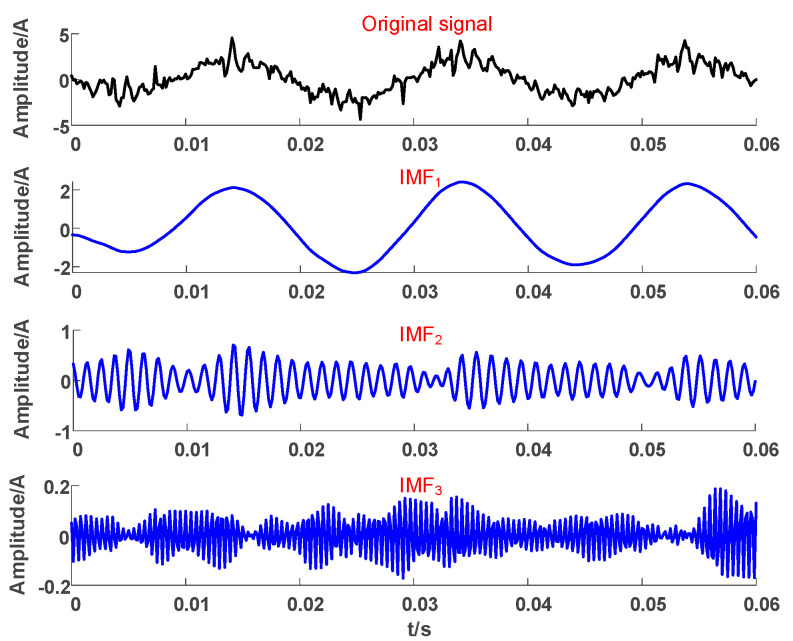
Decomposition results of ZSC.

As shown in Figure 4, the original fault waveform is divided into three modes, and transforming them into the corresponding frequency domain indicates that each mode has actual physical significance: IMF_1_ corresponds to 50 Hz, representing the fundamental frequency component; IMF_2_ corresponds to 750 Hz, representing the high-frequency component; and IMF_3_ corresponds to high-frequency noise signals above 2000 Hz.

**Figure 5:**
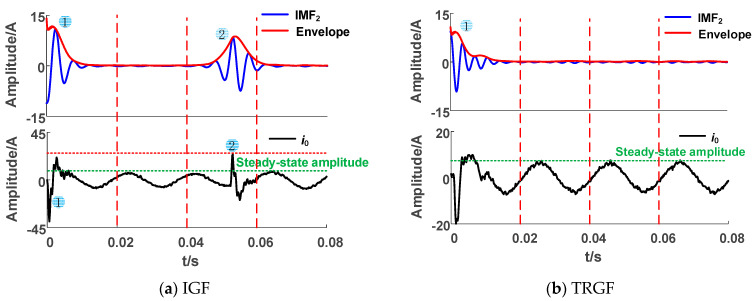
High frequency component envelope lines of actual system faults.

When the grounding fault occurs, the transient high-frequency component changes obviously. As shown in Figure 5a, there are two transient processes in the IGF waveform. Observing the envelope of its high-frequency component IMF_2_, we found that there are two peaks in different periods, which can be used to identify IGFs. The high-frequency component of the transition resistance grounding fault (TRGF) in Figure 5b shows a decreasing oscillation trend, with no second peak appearing.

Criterion 1: Calculating the number of peaks in each period of the characteristic IMFs after fault occurred, we obtained the peak distribution matrix *S* = [*s*_1_ *s*_2_ *s*_3_ *… s*_T_]. If Formula (10) is satisfied, it indicates that there is not only one transient process in the high-frequency component, corresponding to the characteristics of the IGF (Label Ⅰ) and AGF (Label Ⅰ), and the fault with Label Ⅱ has only one transient process.

(10) $$\left\{\begin{array}{l} \Sigma_{S}=s_{1}+s_{2}+\cdots +\cdots s_{T}\\ \Sigma_{S}>1 \end{array}\right.$$

Due to the reignition of the AGF leading to multiple transient processes, Criterion 2 is constructed to distinguish between an IGF and AGF.

### 3.2. Criterion 2

For an AGF, in addition to the first transient process of the fault, there are also multiple arcing and arc-extinguishing states, so the complexity of the waveform is greater than that of the IGF. Permutation entropy can measure the complexity of time series [31]. It introduces permutation when calculating the complexity between reconstructed subsequences. There is a time series *X*(*t*) = {*x*(1), *x*(2), *x*(3),…, *x*(*n*)} of length *n*. We specify an embedding dimension *m* and a time delay τ, and then reconstruct the original sequence as

(11) $$Y=[\begin{array}{c} L(1)\\ L(2)\\ L(t)\\ \vdots \\ L(j) \end{array}]=[\begin{array}{cccc} x(1) & x(1+\tau ) & \cdots & x[1+(m-1)\tau ]\\ x(2) & x(2+\tau ) & \cdots & x[2+(m-1)\tau ]\\ x(t) & x(t+\tau ) & \cdots & x[t+(m-1)\tau ]\\ \vdots & \vdots & \vdots & \vdots \\ x(j) & x(j+\tau ) & \cdots & x[j+(m-1)\tau ] \end{array}]$$

In the above formula, j=n−(m−1)τ, each row *L*(*t*) of *Y* is a reconstruction component, and each reconstruction component is reordered in ascending order:

(12) $$x[i+(k_{1}-1)\tau ]\leq x[i+(k_{2}-1)\tau ]\leq \ldots \leq x[i+(k_{m}-1)\tau ]$$

We obtained the permutation order of each reconstructed component:

(13) $$K_{L\_t}=\{k_{1},k_{2},\ldots ,k_{m}\}$$

The probability of each permutation order is $P_{L\_t}=\frac{Num(K_{L\_t})}{j}$, and the permutation entropy formula is defined as

(14) $$pe=-\sum P_{L\_t}\ln(P_{L\_t}),t=1,2,\ldots ,K_{\sum }$$

$Num(K_{L\_t})$ is the occurrence number of the same order, and $K_{\sum }$ is the type of permutation order, which has at most *m*! permutations, so $K_{\sum }\leq m!$.

Based on the above analysis, we propose detection Criterion 2. Calculate the permutation entropy *pe* of ZSC for each feeder. If $pe\geq pe_{set}$, the fault is judged as an AGF; otherwise, we determine that an IGF has occurred. After a large number of experiments under different fault conditions, we set the $pe_{set}$ for the radial distribution network and the actual system (where the recorder data are obtained from) as 0.9, and the $pe_{set}$ for the unbalanced distribution network as 0.8.

### 3.3. Criterion 3

During the recovery process after the SLGF disappears, the ZSV shows a trend of oscillation and attenuation. In order to calculate this attenuation trend, we define the attenuation degree of ZSV in the *T*-th period after the fault as

(15) $$\alpha_{T}=\frac{\left|U_{0}^{T+1}\right|}{\left|U_{0}^{T}\right|}$$

where $U_{0}^{T}$ and $U_{0}^{T+1}$ are the ZSV amplitudes of the *T*-th and (*T* + 1)-th periods after the fault. Generally, when the ZSV continues to decay to 5% of the amplitude at the time of the fault, the system is considered to be restored.

Criterion 3 is constructed. When the amplitude of the ZSV continues to decay for *N* periods (starting from the *T*-th period), the attenuation set ∂ is $\left\{\alpha_{T+1},\right.\alpha_{T+2},\ldots ,\alpha_{T+N}\}$. If ∂ meets $\left\{\alpha_{t}\in \partial \left|\alpha_{t}\leq 1\right.\right\}$ and the ZSV meets $\left|U_{0}^{T+N}\right|\leq 5\%\left|U_{0}^{1}\right|$, it indicates that a TGF has occurred; otherwise, the fault is judged as a TRGF.

### 3.4. Criterion 4

In the fault analysis in Section 2, we obtained $G_{F}$∝$\left|{\dot{U}}_{\mathrm{ub}}{}^{\prime }-{\dot{U}}_{\mathrm{ub}}\right|$ as well as $G_{F}$∝$1/\left|{\dot{U}}_{A}\right|$. On this basis, $K_{\mathrm{set}}$ is introduced to facilitate the classification of the grounding transition resistance.

(16) $$K_{\mathrm{set}}=\frac{G_{F}}{Y_{0\sum }}=\frac{\left|\Delta {\dot{U}}_{0}\right|}{\left|{\dot{U}}_{A}\right|}$$

where $\Delta {\dot{U}}_{0}={\dot{U}}_{\mathrm{ub}}{}^{\prime }-{\dot{U}}_{\mathrm{ub}}$ is the variation of ZSV, and the transition resistance is negatively correlated with $K_{set}$, so it is also positively correlated with the variation of ZSV. With $K_{set1}$ and $K_{\mathrm{set}2}$ as the boundary, there are three situations:

(17) $$\left\{\begin{array}{l} K_{\mathrm{set}}\leq K_{\mathrm{set}1}\\ K_{\mathrm{set}1}<K_{\mathrm{set}}\leq K_{\mathrm{set}2}\\ K_{\mathrm{set}}>K_{\mathrm{set}2} \end{array}\right.$$

We obtain Criterion 4. If $K_{\mathrm{set}}$ ≤ $K_{\mathrm{set}1}$, the fault is determined as an HIF. If $K_{\mathrm{set}1}$ < $K_{\mathrm{set}}$ ≤ $K_{\mathrm{set}2}$, the fault is determined as an MIF. Otherwise, $K_{\mathrm{set}}$ > $K_{\mathrm{set}2}$, and we judge the fault as an SIF. After extensive experiments under different fault conditions, we set the radial distribution network with $K_{\mathrm{set}1}$ = 0.1 and $K_{\mathrm{set}2}$ = 1, the unbalanced distribution network with $K_{\mathrm{set}1}$ = 0.005 and $K_{\mathrm{set}2}$ = 0.05, and the actual system (where the recorder data are obtained from) with $K_{\mathrm{set}1}$ = 1 and $K_{\mathrm{set}2}$ = 5.

### 3.5. Multi-Type Fault Classification Method

**Figure 6:**
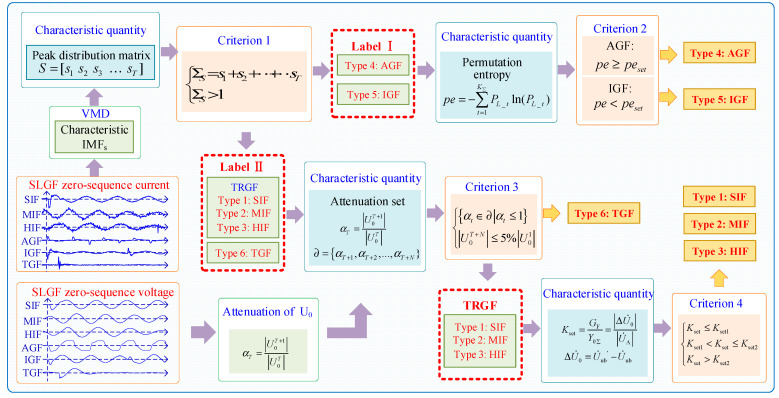
Multilevel fault nature classification process.

We propose a multilevel fault nature classification process as shown in Figure 6. The classification process covers 4 criteria. According to the flow chart of multi-category fault identification, the classification steps are listed as follows.

Steps: (1) The ZSC and ZSV of each fault feeder are taken as the object of feature analysis. The ZSC of all faults is decomposed by VMD, and we extract the frequency characteristics under different spectra and relate them to the time domain.

(2) After calculating the peaks of the feature IMFs, Criterion 1 categorizes AGFs and IGFs with multiple transient processes into a large category.

(3) By using Criterion 2 to calculate the ZSC permutation entropy *pe*, AGFs and IGFs can be distinguished.

(4) After determining attenuation set ∂ and calculating the amplitude of ZSV $\left|U_{0}^{T+N}\right|$ in the last period, the TGF can be distinguished by Criterion 3.

(5) Criterion 4 divides TRGFs into SIFs, MIFs, and HIFs according to the transition resistance.

## 4. Simulation and Test Verification

**Figure 7:**
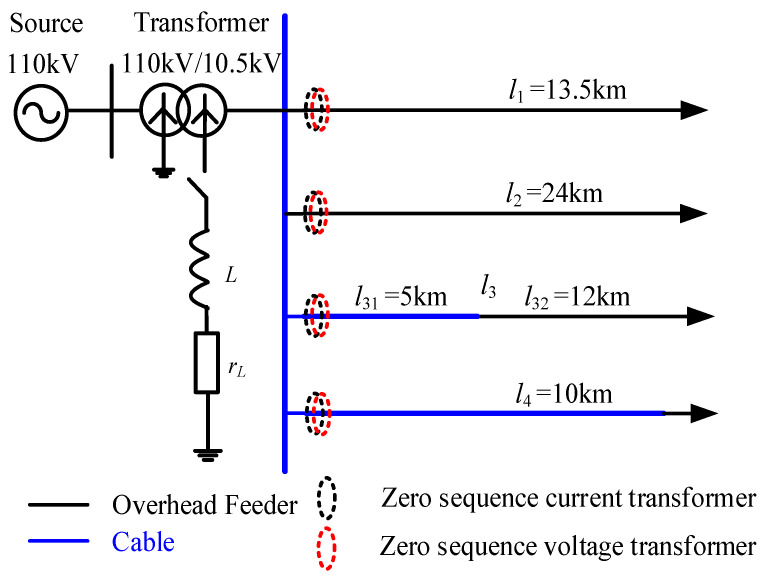
Radial distribution network model.

As shown in Figure 7, we established a 10 kV radial distribution network model in the electromagnetic transient simulation software. It consists of four feeders, among which feeder *l*_1_ and *l*_2_ are overhead lines, feeder *l*_3_ includes a cable and overhead line, and feeder *l*_4_ is a cable. The parameters of the overhead line and cable are shown in Table 1 [32].

### 4.1. Different Fault Condition Test

To verify the universality of the proposed method, different fault conditions were simulated. In Table 2, the different fault feeders, fault transition resistance, and fault types are listed.

**Figure 8:**
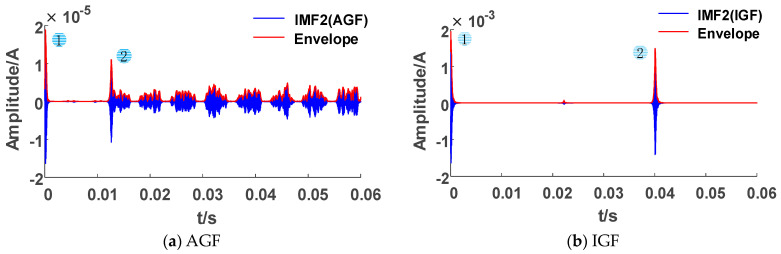
High frequency component envelope lines for radial network faults.

We collected the ZSC and ZSV of faults under different conditions and identified faults by the classification process proposed in Section 2. Firstly, we calculated the number in the peak matrix. As shown in Figure 8, when AGFs and IGFs occur, the number of peaks within the statistical period is greater than 1. However, the transient processes of TGF, SIF, MIF, and HIF faults only occur in the initial stage of the fault, so there is only one peak. The results of Criterion 1 are shown in Table 3, and the faults are preliminarily divided into two labels: Label I and Label II.

Secondly, Criterion 2 is used to calculate the permutation entropy value of each fault feeder in Label I. The permutation entropy *pe* reflects the complexity of the ZSC waveform. In Table 4, we can see that the *pe* values of AGFs are larger than $pe_{set}$ (value is 0.9), while the *pe* values of IGFs are lower. Thus, Type 4 (AGF) and Type 5 (IGF) are distinguished.

**Figure 9:**
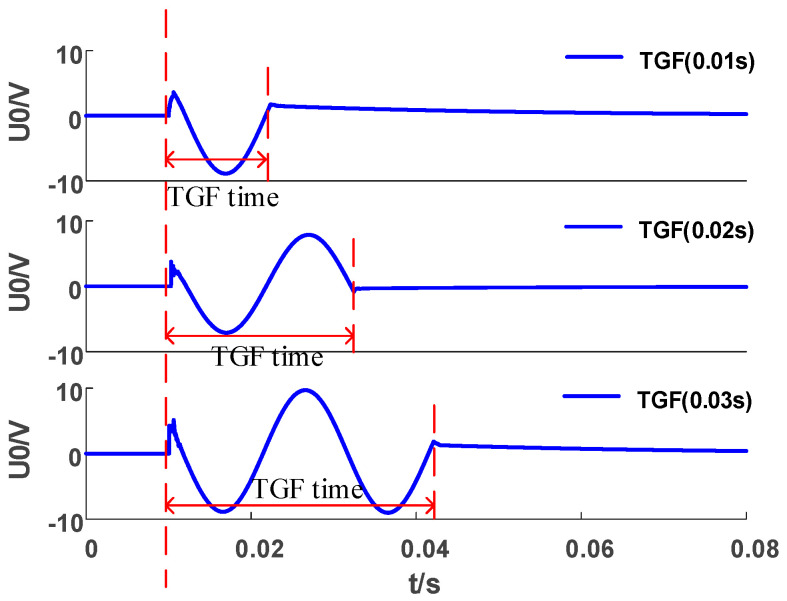
ZSV of TGF fault.

Then, we selected the fault that met Criterion 3 from Label II. Figure 9 shows the change trend of the ZSV during the occurrence of a TGF. It can be seen that when the fault is over, the ZSV continues to decrease. As shown for the TGF in Table 5, the attenuation degree $\alpha_{T}$ of the first four periods is less than 1, and the ZSV value in the fifth period is less than 5% of the initial value. In the TRGF, except for an HIF, the $\alpha_{T}$ of all other faults are greater than 1 within the statistical periods, indicating that the waveform has not decayed. However, when the HIF voltage no longer decays, the ZSV value is too large to meet $\left|U_{0}^{T+N}\right|\leq 5\%\left|U_{0}^{1}\right|$, so it is judged as a TRGF (SIF, MIF, and HIF).

Finally, based on the variation of ZSV and the voltage of the fault phase, we calculated the value of $K_{set}$, which is positively correlated with the transition conductance; then, we compared it with the criterion parameters $K_{\mathrm{set}1}$ (value is 0.1) and $K_{\mathrm{set}2}$ (value is 1) in Criterion 4. The results are shown in Table 6. The SIF meets $K_{\mathrm{set}}$ > $K_{\mathrm{set}2}$, the MIF meets $K_{\mathrm{set}1}$ < $K_{\mathrm{set}}$ ≤ $K_{\mathrm{set}2}$, and the HIF meets $K_{\mathrm{set}}$ ≤ $K_{\mathrm{set}1}$. It can be seen that the order of magnitude of $K_{set}$ decreases with the increase in transition resistance.

### 4.2. Unbalanced Load Test

**Figure 10:**
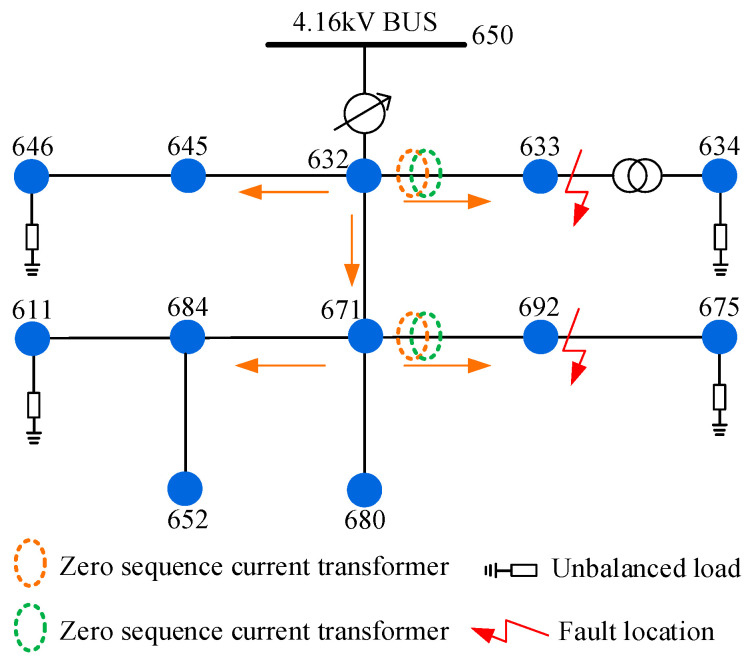
IEEE-13 node distribution network model.

As shown in Figure 10, we established an IEEE-13 node model. Its nominal voltage is 4.16 kV, and it is characterized by short lines, high loads, substation voltage regulators, overhead feeders and underground cables, parallel capacitors, line transformers, and unbalanced loads.

Six types of SLGFs were simulated at nodes 633 and 692, and the ZSC and ZSV were recorded by sensors installed at the heads of the lines. The judgment results are shown in Table 7.

As shown in Table 7 and Table 8, the peak matrix was calculated and determined by Criterion 1, where the sum of the peaks in Label I is higher than 1. Then, the permutation entropy of ZSC in Label I (AGF and IGF) was calculated by Criterion 2. Since the AGF waveform is complex, the *pe* value of the AGF is much larger than that of the IGF.

Table 9 and Table 10 show the classification results of faults included in Label II (SIF, MIF, and HIF). By analyzing the trend of ZSV attenuation degree $\alpha_{T}$, we can check whether the ZSV value at the end of attenuation is less than the 5% of the initial fault value. The fault that meets Criterion 3 is a TGF; otherwise, it is a TRGF. The $K_{set}$ value of the TRGF is compared by Criterion 4, which ranges from high to low in SIFs, MIFs, and HIFs.

## 5. Practical Fault Data Verification

**Figure 11:**
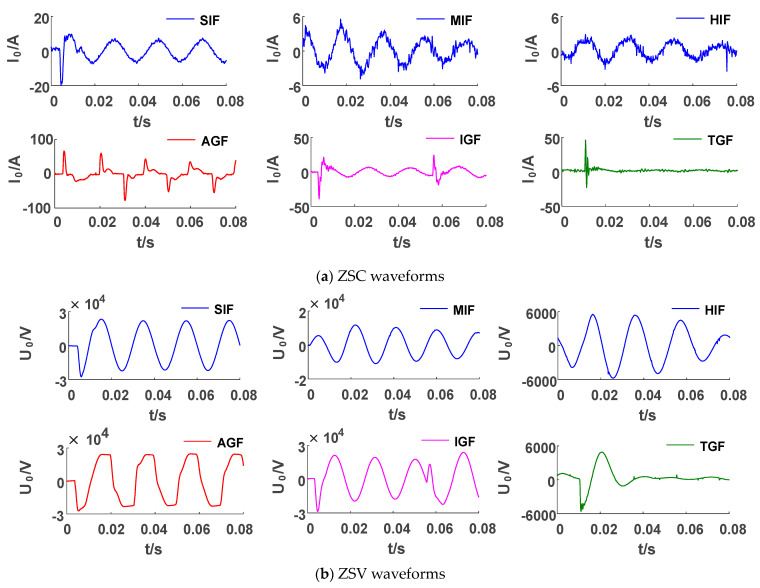
Recorded waveforms of actual SLGFs.

The SLGF data are recorder waveforms of the Xinjiang distribution network in China, covering six fault types, SIF, MIF, HIF, AGF, IGF, and TGF, as shown in Figure 11.

As shown in Figure 11a, when an SIF occurs, the zero sequence current waveform suddenly changes and then returns to a stable state. The comparison of SIFs, MIFs, and HIFs shows that the current amplitude decreases with the increase in transition resistance. The AGF current waveform has obvious extinction–reignition–extinction characteristics and gradually increases to its maximum value. There is an obvious transient process in the initial stage of the IGF current, and it then gradually stabilizes. However, the waveform oscillates again and a second transient process appears. The ZSC waveform oscillates significantly during the TGF occurrence; the ZSC approaches zero after fault recovery.

In the ZSV waveforms shown in Figure 11b, the SIF, MIF, and HIF waveforms are similar to sine waves, and only their amplitudes are different. The AGF waveform has significant distortion, with a saddle-shaped appearance. The IGF voltage also has a second transient process like the current. The ZSV continues to decay to approximately 0 after the TGF disappears.

Table 11 shows the judgment results of the six types of fault waveforms. As can be seen, based on Criterion 1 and Criterion 2, it is possible to preliminarily determine Type 4 (AGF) and Type 5 (IGF); other waveforms that do not belong to these two Types will be classified as Type 6 (TGF) and TRGFs (SIFs, MIFs, and HIFs) based on Criterion 3. Finally, the TRGF is classified as Type 1 (SIF), Type 2 (MIF), and Type 3 (HIF) by Criterion 4. All types of faults are judged correctly.

## 6. Adaptability Analysis

### 6.1. Noise Test

**Figure 12:**
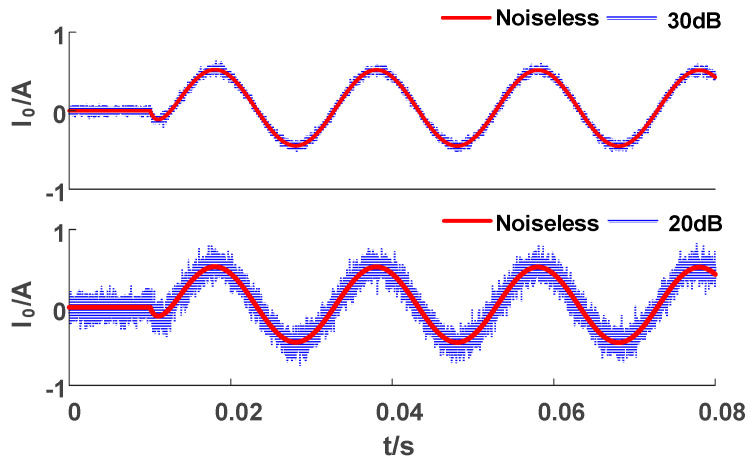
ZSC superimposed with Gaussian white noise.

In practical applications, due to the presence of various disturbances, the quality of the received signal will decrease [33,34,35,36]. In order to simulate the noise that often occurs in practical engineering applications, Gaussian white noise is usually superimposed on the original ZSC signal. As shown in Figure 12, after adding Gaussian white noise, the waveform is distorted. With the decrease in the signal-to-noise ratio (SNR), the distortion degree of the zero sequence current waveform superimposed with 20 dB noise (compared to SNR = 30 dB) is higher.

Table 12 shows the classification results of ZSC waveforms with superimposed 20 dB noise. The AGF and IGF are firstly distinguished by the sum of the peaks and the size of the permutation entropy. Then, as the ZSV of the TGF continuously decays for two cycles and the third cycle returns to a normal voltage value, it is distinguished from a TRGF. Finally, the SIF, MIF, and HIF are distinguished due to their different magnitudes.

### 6.2. Data Loss Test

**Figure 13:**
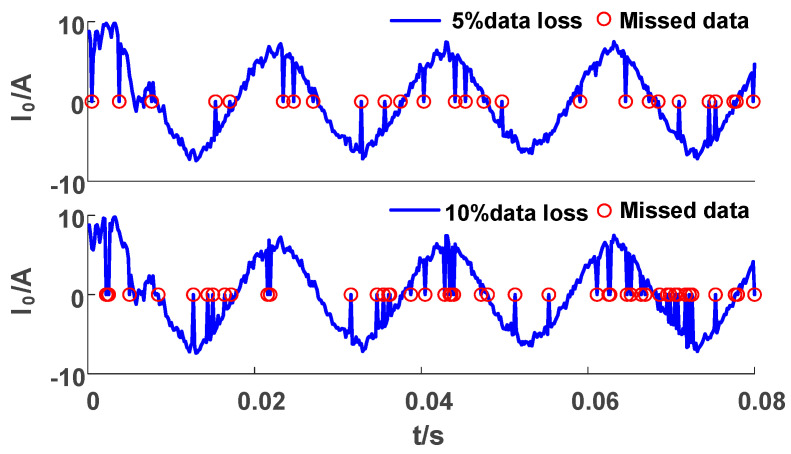
ZSC with random loss.

During the actual operation of the power system, communication may be disturbed, resulting in data loss during transmission. To simulate the above situation, we randomly remove some data and fill in zeros, as shown in Figure 13.

Unlike the impact of the superimposed noise conditions, data loss affects the complexity and amplitude of the waveform. As shown in Table 13, the value of *pe* in Criterion 2 slightly increases, and the $\alpha_{T}$ value in Criterion 3 also changes, but the classification result remains unchanged.

## 7. Conclusions

Aiming at the classification of multiple types of single line-to-ground faults in actual systems, four criteria are proposed by analyzing the same and different features of six types of faults. The proposed method has been validated in radial distribution networks, unbalanced distribution networks, and the realistic recorded data of faulty sensors in an actual system. This is conducive to determining the cause of grounding faults and formulating targeted measures for hidden danger treatment. We obtained the following conclusions.

(1) By deducing the generation mechanism of the zero sequence voltage, we obtained the relationship between the transition conductance, the in ZSV, and the fault phase voltage: the transition conductance is positively correlated with the variation in ZSV and inversely correlated with the fault phase voltage.
(2) After we analyzed and compared the characteristics of various SLGFs, we obtained inductive criteria for the same features and discrimination criteria for different features. Using four criteria to construct a multi-level classification method, the waveforms under different fault conditions were verified, and the results showed that the proposed method has high reliability.
(3) The proposed method judged faults correctly for both balanced and unbalanced topology models. Moreover, the method has good robustness under interference conditions, such as noise effects and data loss. In addition, the validation of actual fault data indicates that the method has certain engineering applicability.

It is necessary to acknowledge the limitations of this work. Some criteria need to accumulate enough data to set the threshold for each different network, which will take a certain time in the actual system. Therefore, in our future work, we intend to deeply investigate the characteristics of the fault itself and reduce the reliance on the data quantity.

## Figures and Tables

**Table 1:** Feeder parameters.

Feeder Types	Phase Sequence	R (Ω/km)	L (mH/km)	C (μF/km)
Overhead feeder	Positive sequence	0.1700	1.2000	0.0097
	Zero sequence	0.2300	5.4800	0.0060
Cable feeder	Positive sequence	0.1930	0.4420	0.1430
	Zero sequence	1.9300	5.4800	0.1430

**Table 2:** Fault setting parameters.

Type	Parameter Value
Fault feeder	*l*_1_, *l*_3_, *l*_4_
Transition resistance/Ω	0.01, 5, 10; 50, 60, 70, 500, 1000, 1500
Arc fault model	Emanuel
Interval time of IGF/s	0.02, 0.04, 0.06
Time of TGF/s	0.01, 0.02, 0.03

**Table 3:** Criterion 1 judgment results for radial network faults.

Type	Fault Feeder	Fault Parameters	Peak Matrix	Number of Peak	Label
AGF	*l*_1_	-	[2 0 0 0]	2 > 1	Ⅰ
	*l*_3_	-	[2 0 0 0]	2 > 1	
	*l*_4_	-	[2 0 0 0]	2 > 1	
IGF	*l*_1_	0.02 s	[1 0 1 0]	2 > 1	
	*l*_3_	0.04 s	[1 0 1 0]	2 > 1	
	*l*_4_	0.06 s	[1 0 0 1]	2 > 1	
TGF	*l*_1_	0.01 s	[1 0 0 0]	1 ≯ 1	Ⅱ
	*l*_3_	0.02 s	[1 0 0 0]	1 ≯ 1	
	*l*_4_	0.03 s	[1 0 0 0]	1 ≯ 1	
SIF	*l*_1_	0.01 Ω	[1 0 0 0]	1 ≯ 1	
	*l*_3_	5 Ω	[1 0 0 0]	1 ≯ 1	
	*l*_4_	10 Ω	[1 0 0 0]	1 ≯ 1	
MIF	*l*_1_	50 Ω	[1 0 0 0]	1 ≯ 1	
	*l*_3_	60 Ω	[1 0 0 0]	1 ≯ 1	
	*l*_4_	70 Ω	[1 0 0 0]	1 ≯ 1	
HIF	*l*_1_	500 Ω	[1 0 0 0]	1 ≯ 1	
	*l*_3_	1000 Ω	[1 0 0 0]	1 ≯ 1	
	*l*_4_	1500 Ω	[1 0 0 0]	1 ≯ 1	

**Table 4:** Criterion 2 judgment results for radial network faults.

Type	Fault Feeder	Fault Parameters	*pe*	Type	Result
AGF	*l*_1_	-	0.9861	4	AGF
	*l*_3_	-	0.9884	4	AGF
	*l*_4_	-	0.9807	4	AGF
IGF	*l*_1_	0.02 s	0.5473	5	IGF
	*l*_3_	0.04 s	0.5182	5	IGF
	*l*_4_	0.06 s	0.3851	5	IGF

Note: *pe* is the permutation entropy of the ZSC waveform.

**Table 5:** Criterion 3 judgment results for radial network faults.

Type	Fault Feeder	Parameters	$\alpha_{T}$	$\left|U_{0}^{T+N}\right|$	Result
TGF	*l*_1_	0.01 s	[0.20 0.53 0.53 0.54]	0.14	TGF
	*l*_3_	0.02 s	[0.18 0.15 0.53 0.53]	0.04	TGF
	*l*_4_	0.03 s	[0.70 1.17 0.53 0.53]	0.22	TGF
SIF	*l*_1_	0.01 Ω	[1.02 1.00 1.00 1.00]	15.63	TRGF
	*l*_3_	5 Ω	[1.02 1.00 1.00 1.00]	8.63	
	*l*_4_	10 Ω	[1.00 1.00 1.00 1.00]	9.36	
MIF	*l*_1_	50 Ω	[1.01 1.00 1.00 1.00]	3.50	
	*l*_3_	60 Ω	[1.01 1.00 1.00 1.00]	2.60	
	*l*_4_	70 Ω	[1.00 1.00 1.00 1.00]	2.65	
HIF	*l*_1_	500 Ω	[1.00 1.00 1.00 1.00]	0.43	
	*l*_3_	1000 Ω	[0.99 1.00 1.00 1.00]	0.20	
	*l*_4_	1500 Ω	[0.99 1.00 1.00 1.00]	0.15	

Note: $\alpha_{T}$ is the attenuation degree of the ZSV waveform, $\left|U_{0}^{T+N}\right|$ is the ZSV amplitudes in the *T*-th period.

**Table 6:** Criterion 4 judgment results for radial network faults.

Type	Fault Feeder	Fault Parameters	$K_{\mathrm{set}}$	Type	Result
SIF	*l*_1_	0.01 Ω	9.6632	1	SIF
	*l*_3_	5 Ω	2.5855	1	SIF
	*l*_4_	10 Ω	3.1688	1	SIF
MIF	*l*_1_	50 Ω	0.7096	2	MIF
	*l*_3_	60 Ω	0.4960	2	MIF
	*l*_4_	70 Ω	0.5105	2	MIF
HIF	*l*_1_	500 Ω	0.0727	3	HIF
	*l*_3_	1000 Ω	0.0330	3	HIF
	*l*_4_	1500 Ω	0.0242	3	HIF

Note: $K_{\mathrm{set}}$ is a defined value that is negatively correlated with the transition resistance.

**Table 7:** Criterion 1 judgment results for unbalanced load network faults.

Type	Fault Location	Fault Parameters	Peak Matrix	Number of Peaks	Label
AGF	633	-	[2 0 0 0]	2 > 1	Ⅰ
	692	-	[2 0 0 0]	2 > 1	
IGF	633	0.02 s	[1 1 0 0]	2 > 1	
	692	0.04 s	[1 0 1 0]	2 > 1	
TGF	633	0.01 s	[1 0 0 0]	1 ≯ 1	Ⅱ
	692	0.03 s	[1 0 0 0]	1 ≯ 1	
SIF	633	0.01 Ω	[1 0 0 0]	1 ≯ 1	
	692	5 Ω	[1 0 0 0]	1 ≯ 1	
MIF	633	50 Ω	[1 0 0 0]	1 ≯ 1	
	692	60 Ω	[1 0 0 0]	1 ≯ 1	
HIF	633	500 Ω	[1 0 0 0]	1 ≯ 1	
	692	1000 Ω	[1 0 0 0]	1 ≯ 1	

**Table 8:** Criterion 2 judgment results for unbalanced load network faults.

Type	Fault Location	Fault Parameters	*pe*	Type	Result
AGF	633	-	0.8202	4	AGF
	692	-	0.8633	4	AGF
IGF	633	0.02 s	0.4340	5	IGF
	692	0.04 s	0.4375	5	IGF

Note: *pe* is the permutation entropy of the ZSC waveform.

**Table 9:** Criterion 3 judgment results for unbalanced load network faults.

Type	Fault Location	$\alpha_{T}$	$\left|U_{0}^{T+N}\right|$	Result
TGF	633	[0.58 0.99 1.13 1.07]	$\left|U_{0}^{3}\right|$ = 0.2147	TGF
	692	[0.40 0.64 1.22 1.08]	$\left|U_{0}^{3}\right|$ = 0.1285	TGF
SIF	633	[1.03 1.00 1.01 1.02]	$\left|U_{0}^{5}\right|$ = 3.1642	TRGF
	692	[1.04 1.00 1.07 1.06]	$\left|U_{0}^{5}\right|$ = 0.3523	
MIF	633	[1.01 1.00 1.11 1.06]	$\left|U_{0}^{5}\right|$ = 0.2697	
	692	[1.01 1.00 1.16 1.07]	$\left|U_{0}^{5}\right|$ = 0.1206	
HIF	633	[1.00 1.00 1.13 1.06]	$\left|U_{0}^{5}\right|$ = 0.2454	
	692	[1.00 1.00 1.20 1.08]	$\left|U_{0}^{5}\right|$ = 0.1001	

Note: $\alpha_{T}$ is the attenuation degree of the ZSV waveform, $\left|U_{0}^{T+N}\right|$ is the ZSV amplitude in the *T*-th period.

**Table 10:** Criterion 4 judgment results for unbalanced load network faults.

Type	Fault Feeder	Fault Parameters	$K_{\mathrm{set}}$	Type	Result
SIF	*l*_1_	0.01 Ω	4.3610	1	SIF
	*l*_4_	10 Ω	0.1095	1	SIF
MIF	*l*_1_	50 Ω	0.0125	2	MIF
	*l*_4_	70 Ω	0.0097	2	MIF
HIF	*l*_1_	200 Ω	0.0013	3	HIF
	*l*_4_	1000 Ω	0.0006	3	HIF

Note: $K_{\mathrm{set}}$ is a defined value that is negatively correlated with the transition resistance.

**Table 11:** Judgment results of actual data.

Type	Peak Matrix	*pe*	$\alpha_{T}$	$\left|U_{0}^{T+N}\right|$	$\left|U_{0}^{T+N}\right|$	$K_{\mathrm{set}}$	Result	Correct?
AGF	[2 2 2 2]	0.9136	-	-	-	-	4 → AGF	√
	[1 2 1 2]	0.9467	-	-	-	-	4 → AGF	√
	[1 2 2 2]	0.9060	-	-	-	-	4 → AGF	√
IGF	[1 0 1 0]	0.7826	-	-	-	-	5 → IGF	√
	[1 0 1 0]	0.7268	-	-	-	-	5 → IGF	√
	[1 0 0 1]	0.7446	-	-	-	-	5 → IGF	√
TGF	[1 0 0 0]	-	[0.19 0.47 1.06 1.05]	$\left|U_{0}^{3}\right|$ = 272.40	$\left|U_{0}^{3}\right|\leq 5\%\left|U_{0}^{1}\right|$	-	6 → TGF	√
	[1 0 0 0]	-	[0.19 0.78 1.00 1.02]	$\left|U_{0}^{4}\right|$ = 301.30	$\left|U_{0}^{4}\right|\leq 5\%\left|U_{0}^{1}\right|$	-	6 → TGF	√
	[1 0 0 0]	-	[0.90 0.43 1.00 1.07]	$\left|U_{0}^{4}\right|$ = 350.56	$\left|U_{0}^{4}\right|\leq 5\%\left|U_{0}^{1}\right|$	-	6 → TGF	√
SIF	[1 0 0 0]	-	[0.92 1.00 1.02 1.00]	$\left|U_{0}^{3}\right|$ = 15,413.06	$\left|U_{0}^{3}\right|>5\%\left|U_{0}^{1}\right|$	10.01	1 → SIF	√
	[1 0 0 0]	-	[0.99 1.02 1.01 1.00]	$\left|U_{0}^{3}\right|$ = 15,532.50	$\left|U_{0}^{3}\right|>5\%\left|U_{0}^{1}\right|$	11.95	1 → SIF	√
	[1 0 0 0]	-	[0.94 0.99 0.96 0.94]	$\left|U_{0}^{5}\right|$ = 16,647.74	$\left|U_{0}^{3}\right|>5\%\left|U_{0}^{1}\right|$	7.55	1 → SIF	√
MIF	[1 0 0 0]	-	[0.97 0.87 0.85 0.77]	$\left|U_{0}^{5}\right|$ = 4199.69	$\left|U_{0}^{5}\right|>5\%\left|U_{0}^{1}\right|$	1.94	2 → MIF	√
	[1 0 0 0]	-	[0.92 1.02 0.99 0.99]	$\left|U_{0}^{2}\right|$ = 10,026.04	$\left|U_{0}^{2}\right|>5\%\left|U_{0}^{1}\right|$	1.79	2 → MIF	√
	[1 0 0 0]	-	[0.93 1.02 1.00 0.99]	$\left|U_{0}^{2}\right|$ = 10,102.80	$\left|U_{0}^{2}\right|>5\%\left|U_{0}^{1}\right|$	1.77	2 → MIF	√
HIF	[1 0 0 0]	-	[1.02 0.83 0.50 0.50]	$\left|U_{0}^{5}\right|$ = 767.51	$\left|U_{0}^{5}\right|>5\%\left|U_{0}^{1}\right|$	0.50	3 → HIF	√
	[1 0 0 0]	-	[0.94 0.93 0.92 0.87]	$\left|U_{0}^{5}\right|$ = 882.50	$\left|U_{0}^{5}\right|>5\%\left|U_{0}^{1}\right|$	0.86	3 → HIF	√
	[1 0 0 0]	-	[0.93 0.91 0.86 0.27]	$\left|U_{0}^{5}\right|$ = 514.25	$\left|U_{0}^{5}\right|>5\%\left|U_{0}^{1}\right|$	0.81	3 → HIF	√

Note: *pe* is the permutation entropy of the ZSC waveform, $\alpha_{T}$ is the attenuation degree of the ZSV waveform, $\left|U_{0}^{T+N}\right|$ is the ZSV amplitude in the *T*-th period, *K*_set_ is a defined value that is negatively correlated with the transition resistance.

**Table 12:** Judgment results under 20 dB noise.

Type	Peak Matrix	*pe*	$\alpha_{T}$	$\left|U_{0}^{T+N}\right|$	$\left|U_{0}^{T+N}\right|$	$K_{\mathrm{set}}$	Result	Correct?
AGF	[2 2 2 2]	0.9118	-	-	-	-	4 → AGF	√
IGF	[1 0 1 0]	0.7412	-	-	-	-	5 → IGF	√
TGF	[1 0 0 0]	-	[0.19 0.47 1.06 1.05]	$\left|U_{0}^{3}\right|$ = 272.40	$\left|U_{0}^{3}\right|\leq 5\%\left|U_{0}^{1}\right|$	-	6 → TGF	√
SIF	[1 0 0 0]	-	[0.92 1.00 1.02 1.00]	$\left|U_{0}^{3}\right|$ = 15,403.64	$\left|U_{0}^{3}\right|>5\%\left|U_{0}^{1}\right|$	10.01	1 → SIF	√
MIF	[1 0 0 0]	-	[0.97 0.87 0.85 0.77]	$\left|U_{0}^{5}\right|$ = 4199.67	$\left|U_{0}^{5}\right|>5\%\left|U_{0}^{1}\right|$	1.94	2 → MIF	√
HIF	[1 0 0 0]	-	[1.02 0.83 0.50 0.50]	$\left|U_{0}^{5}\right|$ = 767.51	$\left|U_{0}^{5}\right|>5\%\left|U_{0}^{1}\right|$	0.50	3 → HIF	√

Note: *pe* is the permutation entropy of the ZSC waveform, $\alpha_{T}$ is the attenuation degree of the ZSV waveform, $\left|U_{0}^{T+N}\right|$ is the ZSV amplitude in the *T*-th period, $K_{\mathrm{set}}$ is a defined value that is negatively correlated with the transition resistance.

**Table 13:** Judgment results under 5% data loss.

Type	Peak Matrix	*pe*	$\alpha_{T}$	$\left|U_{0}^{T+N}\right|$	$\left|U_{0}^{T+N}\right|$	$K_{\mathrm{set}}$	Result	Correct?
AGF	[2 2 2 2]	0.9369	-	-	-	-	4 → AGF	√
IGF	[1 0 1 0]	0.8170	-	-	-	-	5 → IGF	√
TGF	[1 0 0 0]	-	[0.18 0.47 1.07 1.05]	$\left|U_{0}^{3}\right|$ = 261.97	$\left|U_{0}^{3}\right|\leq 5\%\left|U_{0}^{1}\right|$	-	6 → TGF	√
SIF	[1 0 0 0]	-	[0.93 0.99 1.01 1.02]	$\left|U_{0}^{3}\right|$ = 14,955.07	$\left|U_{0}^{3}\right|>5\%\left|U_{0}^{1}\right|$	10.01	1 → SIF	√
MIF	[1 0 0 0]	-	[0.97 0.88 0.85 0.78]	$\left|U_{0}^{5}\right|$ = 4116.56	$\left|U_{0}^{5}\right|>5\%\left|U_{0}^{1}\right|$	1.94	2 → MIF	√
HIF	[1 0 0 0]	-	[1.02 0.82 0.51 0.50]	$\left|U_{0}^{5}\right|$ = 753.20	$\left|U_{0}^{5}\right|>5\%\left|U_{0}^{1}\right|$	0.50	3 → HIF	√

Note: *pe* is the permutation entropy of the ZSC waveform, $\alpha_{T}$ is the attenuation degree of the ZSV waveform, $\left|U_{0}^{T+N}\right|$ is the ZSV amplitude in the *T*-th period, *K*_set_ is a defined value that is negatively correlated with the transition resistance.

## Data Availability

The data presented in this study are available on request from the corresponding author. The data are not publicly available due to the need to use some of the data in this paper in subsequent research.

## Abbreviations

SLGF	Single line-to-ground fault
ZSV	Zero sequence voltage
WT	Wavelet transform
ZSC	Zero sequence current
VMD	Variational mode decomposition
EMD	Empirical mode decomposition
CEEMDAN	Complete ensemble empirical mode decomposition with adaptive noise
IMF	Intrinsic mode function
CNN	Convolutional neural network
TRGF	Transition resistance grounding fault
SIF	Small-impedance fault
MIF	Medium-impedance fault
HIF	High-impedance fault
AGF	Arc grounding fault
IGF	Intermittent grounding fault
TGF	Transient grounding fault
